# Detection of Computer Graphics Using Attention-Based Dual-Branch Convolutional Neural Network from Fused Color Components

**DOI:** 10.3390/s20174743

**Published:** 2020-08-22

**Authors:** Peisong He, Haoliang Li, Hongxia Wang, Ruimei Zhang

**Affiliations:** 1 College of Cybersecurity, Sichuan University, Chengdu 610065, China; gokeyhps@scu.edu.cn (P.H.); ruimeizhang163@163.com (R.Z.); 2 School of Electrical and Electronic Engineering, Nanyang Technological University, Singapore 637553, Singapore; lihaoliang@ntu.edu.sg

**Keywords:** image forensics, computer graphics, convolutional neural network, attention-based fusion, fused color components

## Abstract

With the development of 3D rendering techniques, people can create photorealistic computer graphics (CG) easily with the advanced software, which is of great benefit to the video game and film industries. On the other hand, the abuse of CGs has threatened the integrity and authenticity of digital images. In the last decade, several detection methods of CGs have been proposed successfully. However, existing methods cannot provide reliable detection results for CGs with the small patch size and post-processing operations. To overcome the above-mentioned limitation, we proposed an attention-based dual-branch convolutional neural network (AD-CNN) to extract robust representations from fused color components. In pre-processing, raw RGB components and their blurred version with Gaussian low-pass filter are stacked together in channel-wise as the input for the AD-CNN, which aims to help the network learn more generalized patterns. The proposed AD-CNN starts with a dual-branch structure where two branches work in parallel and have the identical shallow CNN architecture, except that the first convolutional layer in each branch has various kernel sizes to exploit low-level forensics traces in multi-scale. The output features from each branch are jointly optimized by the attention-based fusion module which can assign the asymmetric weights to different branches automatically. Finally, the fused feature is fed into the following fully-connected layers to obtain final detection results. Comparative and self-analysis experiments have demonstrated the better detection capability and robustness of the proposed detection compared with other state-of-the-art methods under various experimental settings, especially for image patch with the small size and post-processing operations.

## 1. Introduction

**Figure 1:**
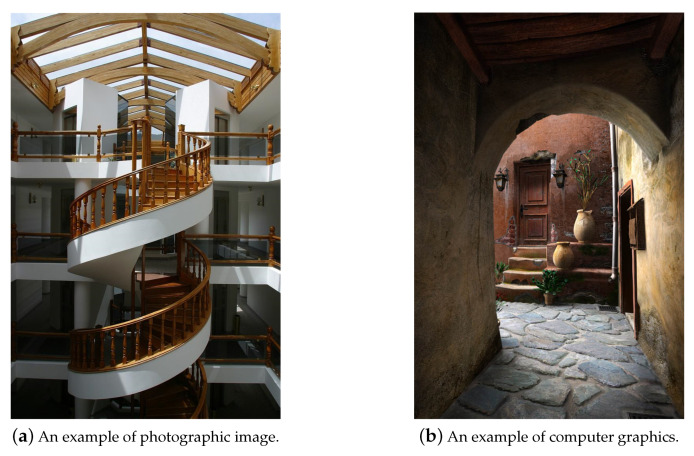
Examples of computer graphics and a photographic image.

In the past few years, with the rapid development of the computer graphics (CGs) technique, the industries of films and video games have benefited from its powerful generation capability of realistic 3D images, which are more and more difficult to be distinguished by human eyes. In Figure 1, we present some examples of CGs and a photographic image (referred to as PG). On the other hand, the forgers can create fake news in social media more efficiently by exploiting advanced 3D graphics rendering software. The abuse of photorealistic CGs has threatened the authenticity and integrity of digital images, especially in academic, medical and forensics applications. Therefore, the detection of CGs has drawn the attention of experts in the field of image forensics.

For the last decade, several detection methods of CGs have been proposed successfully which base on the fact that the generation processes of CGs (e.g., design, geometry and rasterizer) and PGs (e.g., processing modules within the camera device) are different. In the early stage of the research about detecting CGs, researchers proposed plenty of hand-crafted features and combined with machine learning algorithms (e.g., Support Vector Machine) to obtain results. In recent years, deep learning techniques have been applied in several fields successfully (e.g., computer vision) and also adopted to identify CGs. The deep learning-based methods perform better than hand-crafted feature-based methods in most cases. In addition, deep learning-based methods can extract hierarchical representations from training samples automatically instead of making an effort to design hand-crafted features which is time-consuming and requires professional knowledge. In the field of image forensics [1,2,3,4], the reliable detection capability of forensics attributes (e.g., the traces left by the generation process of CG) in small image patches is still a challenging and significant task, which is very useful in the further forensics analysis, such as the detection of tampering and splicing in local regions. However, most of existing CG identification methods suffer from a distinct performance drop to small image patches and become unreliable with post-processing operations. It is due to the information in a small image patch becoming limited, making it is harder to extract reliable statistics. To overcome the above-mentioned problem, in this paper, we design a detection scheme of CGs based on an attention-based dual-branch convolutional neural network (AD-CNN) from fused color components. In the first step, raw RGB components and their corresponding blurred versions of the input sample are stacked together in channel-wise (early fusion strategy) as the input for AD-CNN, where Gaussian low-pass filter is used to conduct the blurring operation. AD-CNN contains two branches that have an identical shallow CNN structure except that the first convolutional layer in each branch has different kernel sizes. The output features from two branches are jointly optimized by the attention-based fusion module and followed by several fully-connected layers to obtain the final detection result.

The main contributions of this work are summarized as follows:

- We proposed the blurred RGB components using Gaussian low-pass filter and stacked them with the raw RGB components of input samples in channel-wise (early fusion strategy) to help the neural network learn more generalized representations and achieve better robustness against post-processing operations.
- Inspired by Inception module [5], we designed a novel neural network architecture which consists of two branches initialized with different convolutional kernels aiming to learn low-level forensics traces in multi-scale. The deep representations from different branches are then jointly optimized by the attention-based fusion module to get the detection result.
- Comparative and self-analysis experiments have been conducted to evaluate the performance of the proposed method under various settings. Our proposed detection scheme has achieved better and more robust detection results compared with state-of-the-art methods, especially for image patches with small sizes (e.g., 32 × 32 and 64 × 64) and common post-processing operations.

The rest of the paper is organized as follows: Section 2 briefly introduces the related works. In Section 3, the motivation and details of the proposed detection scheme are presented. In Section 4, the proposed method is validated and compared with state-of-the-art methods. Section 5 draws the conclusion.

## 2. Related Works

Existing methods for CG identification can be divided into two categories based on how to extract detection features, including hand-crafted feature-based and deep learning-based methods. For hand-crafted feature-based methods, the researchers designed a set of detection features according to the statistical differences between PGs and CGs in terms of color, texture and other physical characteristics. On the other hand, for deep learning-based methods, hierarchical representations of PGs and CGs were extracted from the training samples automatically.

### 2.1. Hand-Crafted Feature-Based Methods

For the first category, the researchers designed detection features in both pixel domain and transformation domain considering the physical differences of the generation processes between CGs and PGs. T.T. Ng et al. [6] proposed a set of physics-motivated features to describe the abnormally physical properties of CGs, in terms of object geometry, illumination condition and capture device. Pan et al. [7] designed the detection feature using fractal geometry to model abnormal traces of color and coarseness in CGs. In addition, the advanced statistics were leveraged to construct detection features about color, texture and shape, such as [8,9,10,11]. Other researchers found out that the clues in the transformation domain are also efficient to distinguish CGs and PGs. Farid and Lyu [12,13] first applied the high-order statistics of wavelet coefficients to expose CGs. In addition, the statistics of coefficients in other transformation domains were also exploited, such as quaternion wavelet domain in [14] and ridgelet/contourlet domains in [15].

### 2.2. Deep Learning-Based Methods

More recently, deep learning techniques have been applied in several areas, such as computer vision [16]. The hierarchical learning capability of convolutional neural network (CNN) is also suitable to model the distinguishable patterns between CGs and PGs [17]. Yu et al. [18] proposed the prior work of leveraging deep neural network to identify CGs, where the authors constructed a conventional CNN architecture to conduct the detection. In [19], the authors proposed a detection framework, which applied a set of learned convolutional filters and several statistical feature extractors to obtain deep representations. Then, deep representations are fed into the multi-layer perceptron to get the final result. Instead of feeding image samples into CNN directly, Yao et al. [20] used three types of high-pass filters to extract sensor pattern noise as the inputs for a 5-layer CNN. This network can achieve a promising performance when the input image has a relatively large size. Different from applying the fixed and manually designed filers as preprocessing, Quan et al. designed a CNN initialized with a trainable convolutional layer in [21]. This network had a promising detection performance for image patches with different resolutions. In addition, the local-to-global strategy based on major voting is used to get the detection result of a full-size image. He et al. [22] designed a hybrid neural network that combines convolutional and recurrent neural networks to model both local and global statistics of CGs and PGs. In [23], Zhang et al. proposed a CNN architecture that contains the hybrid correlation modules to exploit distinguishable channel and pixel correlation between CGs and PGs.

## 3. The Proposed Method

**Figure 2:**
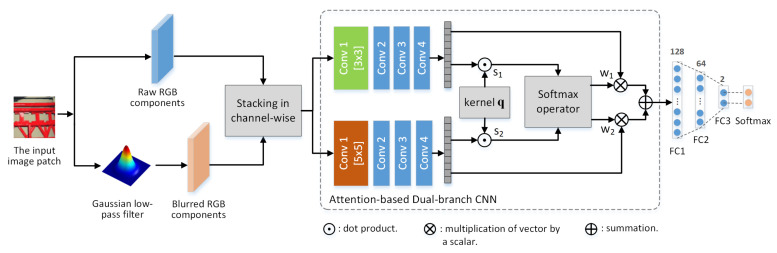
The pipeline of the proposed detection framework.

In this work, we propose an attention-based dual-branch CNN to conduct CG identification, where raw RGB components and their blurred versions are stacked together in channel-wise as the input. The detection framework is presented in Figure 2. The details of the proposed method are presented in this section.

### 3.1. Motivation

The render engine of computer graphics can generate 2D images from 3D scenes by transforming the primitives (e.g., lines and polygons) into raster image representations, such as pixels, where the main steps of rendering include vertex processing, primitive processing, rasterization and fragment processing [24]. All of the above-motioned steps in the render engine are conducted by the computer. On the other hand, the camera device used to capture the photographic images has a quite different generation process which consists of several signal processing operations, such as color filter array, gamma correction and so on [25]. In addition, due to the limitation of computational and memory costs, a representation for fractal surfaces of real-world objects is hard to be obtained perfectly by the render engine. In summary, different generation/capture processes have left distinguishable traces between CGs and PGs in terms of color and texture.

As mentioned in Section 2, deep neural network has been applied to identify CGs successfully. However, most of the existing methods suffer from the distinct performance drop when samples are image patches with the small size, and become unreliable with common post-processing operations. It is because the information within each small image patch is limited and contents among different small image patches are diverse, which is harder to extract robust statistics. This limitation motivates us to develop the more advanced data fusion strategy and network architecture to learn more generalized representations for CG identification.

### 3.2. Preprocessing

In the proposed detection scheme, early fusion strategy is applied to fuse the raw RGB components and their blurred versions as the input of AD-CNN.

#### 3.2.1. Raw RGB Component

In previous works, plenty of deep learning-based methods applied the RGB image patches as the inputs for the neural network directly. In our work, for an input image patch *I*, its three color channels (R, G, B) are considered as raw RGB components $\left\{I_{R},I_{G},I_{B}\right\}\in R^{M\times N\times 3}$, where M×N denotes the size of *I*. This type of components contains information of both color and texture.

#### 3.2.2. Blurred RGB Components

To overcome the above-mentioned problems, the suitable preprocessing operation is necessary, especially when the scale of training samples for the forensics task is relatively limited. We design the blurred version of the raw RGB components (referred to as blurred RGB components) to help the neural network explore more generalized patterns instead of only focusing on local details. More specifically, the high-frequency components of digital images are more sensitive to common post-processing operations (e.g., resizing, noise addition and so on) compared with low-frequency components. The blurred RGB components are generated by blurring the raw RGB components with the Gaussian low-pass filter. More specifically, $\left\{I_{R},I_{G},I_{B}\right\}$ are filtered by the Gaussian low-pass filter *G* individually, where the size of Gaussian low-pass filter is set as 3×3. The blur operation can be formulated as follows:

(1) $$F_{c}=I_{c}*G$$

where “*” denotes the convolutional operation in the spatial domain and $F_{c}$ denotes the blurred RGB components, where c∈{R,G,B}.

#### 3.2.3. Early Fusion Strategy to Generate the Input

We consider a early fusion strategy in our proposed method, where raw RGB components and their blurred versions are stacked together (channel-wise concatenation) as the input ($F=\left\{I_{R},I_{G},I_{B},F_{R},F_{G},F_{B}\right\}\in R^{M\times N\times 6}$) for the network. After the fused color components fed into the network, the computation process of the first convolutional layer can be formulated as Equation (2):

(2) $$H_{j}^{\left(1\right)}=\sum_{l=1}^{L}H_{l}^{\left(0\right)}*W_{lj}^{\left(1\right)}+b_{j}^{\left(1\right)}$$

where “*” denotes the convolutional operation. $H_{l}^{\left(0\right)}$ denotes the *l*-th component from the fused color components F. $H_{j}^{\left(1\right)}$ denotes the *j*-th output feature map from the first convolutional layer. $W_{lj}^{\left(1\right)}$ is the *l*-th channel in the *j*-th convolution kernel of the first convolutional layer. According to Equation (2), it can be observed that the early fusion strategy can make the convolutional layer consider both the spatial-wise and channel-wise correlation between different color components (within or cross raw RGB components and their blurred version). This strategy is efficient to help the network learn a more robust representation instead of overfitting the local details in the optimization process.

### 3.3. The Attention-Based Dual-Branch CNN

The convolutional neural network has been proved to be efficient to learn hierarchical representations from image data, which has been applied in several fields successfully, such as computer vision [16] and multimedia forensics [26]. In this work, we proposed an attention-based dual-branch CNN (referred to as AD-CNN) to learn robust deep representations from fused color components.

#### 3.3.1. Dual-Branch Convolutional Neural Network

As mentioned in Section 3.1, the generation processes of PGs and CGs are different, which can leave distinguishable clues for CG identification. To learn deep representation from the fused color components, we need to design the network architecture carefully. Some ideas are borrowed from one of the typical CNN architectures, namely Inception module [5], where CNN contains different branches (e.g., dual-branch in our network) and each branch equips with different kernel sizes (e.g., 3 × 3 and 5 × 5 in the first convolutional layer). This architecture allows the network to model both local patterns by smaller convolutions and high abstracted patterns via larger convolutions. The weights of each branch do not share with the other branch. Except for the first convolutional layer, the rest of the network structures are identical in two branches, as shown in Table 1. Each branch contains 4 convolutional modules, where each module contains a convolutional layer, a batch normalization layer, a non-linear activation layer and a pooling layer. The output features of two branches $f_{i}\in R^{128\times 1}$, where i∈{1,2}, are fed into the following attention-based fusion module. It should be noted that we did not consider the more complicated connections in our network, such as shortcut connection in Residual Network [27], since the task of CG identification pays more attention to low-level forensics traces instead of high-level concepts about image contents.

#### 3.3.2. The Attention-Based Fusion Module

Fusion technique is always applied to achieve better performance compared with individual features, which has been applied in several applications, such as multimedia forensics [28]. Since the output features from different branches (with 3 × 3 and 5 × 5 kernel sizes in the first convolutional layer) may perform different importance for identifying CGs, the fusion of these two kinds of output features should be adaptive and efficient. In our proposed network, an attention-based fusion module is leveraged to fuse the output features from each branch. The fusion process of this module can be formulated as follows:

(3) $$f_{c}=\sum_{i=1}^{K}w_{i}f_{i}$$

where $w_{i}$ denotes the weights and $f_{i}$ denotes the individual feature. In this work, K=2, including $f_{1}$ and $f_{2}$. Instead of learning the weights $w_{i}$ directly, the attention-based fusion module learns a kernel q, where q has the same dimension as $f_{i}$ and computes the significance $s_{i}$ of each individual feature, namely $s_{i}=q^{T}f_{i}$. To obtain the weights $w_{i}$ from significance values $s_{i}$ and ensure $\sum_{i}w_{i}=1$, the softmax operation is applied to $s_{i}$ as follows:

(4) $$w_{i}=\frac{e^{s_{i}}}{\sum_{j}e^{s_{j}}}$$

This attention-based fusion module is convenient to be embedded in our proposed network and optimized in an end-to-end manner. Then, the attention-based fusion module is followed by three fully-connected layers (referred to as FC1, FC2 and FC3), where the numbers of neurons are 128, 64 and 2, respectively. The non-linear activation applied in FC1 and FC2 is ReLU while the softmax activation used in the last fully-connected layer (FC3). The cross-entropy loss is applied to optimize the network parameters.

### 3.4. Obtain the Final Detection Result

For a given image patch, after the preprocessing operation, we feed it into the trained AD-CNN network and obtain the output vector $\left[p_{1},p_{2}\right]$, where $p_{1}$ and $p_{2}$ denote the probabilities of the input sample belonging to CGs and PGs, respectively. In this work, $p_{1}$ is applied as the detection score and compared with the threshold $T_{f}$ (0.5). If $p_{1}>T_{f}$, the input sample is classified as the image patch of CGs. Otherwise, it is classified as the image patch of PGs.

## 4. Experimental Results

In this section, we conduct experiments on the proposed method and comparisons with state-of-the-art methods. The influence of different network structures is also analyzed.

### 4.1. Dataset

**Figure 3:**
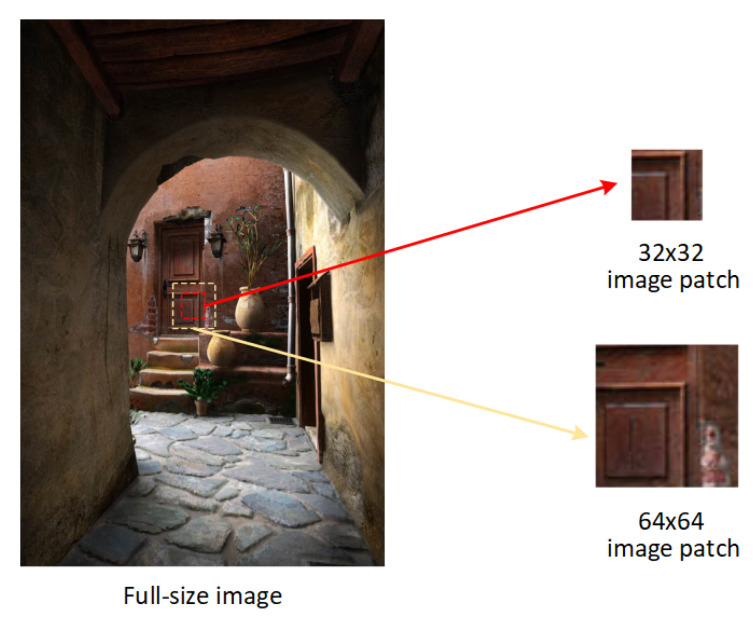
An example of image patches with the small size.

In experiments, the dataset of CGs and PGs in [22] is used to conduct performance evaluation. The CGs in this dataset are collected from the Internet, which are created by more than 50 types of CG rendering software and have various image contents. On the other hand, the PGs in this dataset are captured by different camera devices in both indoor and outdoor scenes with different illumination conditions. There are 6800 CGs and 6800 PGs in this dataset, whose resolutions vary from 266 × 199 to 2048 × 3200. In Figure 1, we present some examples of CG and PG in the dataset [22]. To investigate the detection performance for image patch with the small size b×b (b= 32 or 64), the b×b central region of each image is cropped as a sample. Then, two sets are obtained, including $S_{32\times 32}$ and $S_{64\times 64}$. Samples in $S_{32\times 32}$ and $S_{64\times 64}$ are 32×32 and 64×64 image patches respectively. An example of image patches with the small size is presented in Figure 3. For each set, 6800 pairs of patches from CGs and PGs are randomly split into two parts with the ratio 4:1 for training and testing phases. The part for training is further divided into two subsets with the ratio 9:1 as the training subset and the validation subset to optimize the network weights.

### 4.2. Experimental Settings

In this subsection, we present experimental settings in detail for both evaluation metrics and hyperparameters.

#### 4.2.1. Evaluation Metrics

In experiments, the detection accuracy is used as the evaluation metric, which can be defined as follows:

(5) $$Acc=\frac{TP+TN}{P+N}\times 100\%$$

where *P* and *N* denote the total number of positive samples and negative samples, respectively. In this work, image patches obtained from CGs and PGs are positive and negative samples, respectively. TP and TN are the amount of the positive and negative samples which are classified correctly. True positive rate ($TPR=\frac{TP}{P}\times 100\%$) and true negative rate ($TNR=\frac{TN}{N}\times 100\%$) are also applied to evaluate the detection performance.

#### 4.2.2. Hyperparameters

The weights of the network are optimized with Adam algorithm [29] where the size of a mini-batch is set as 128. The parameters $\beta_{1}$ and $\beta_{2}$ are set as 0.9 and 0.99, respectively. The initial learning rate is set as 0.0005 and is decreased by the factor 0.5 every 20 epochs. The optimal model is obtained based on the performance evaluation on the validation set and the maximum number of epochs is set as 150. The weights of convolutional kernels are initialized based on the method in [30] while the values of biases are set as 0 in the initial stage. The Gaussian low-pass filter used to generate the blurred RGB components is implemented by the function GaussianBlur() in the library OpenCV, where the kernel size is set as 3 and other parameters are set as default. Keras is used to implement the proposed method on the device equipped with the CPU Intel Core i7-8700K and the GPU RTX 2080Ti.

### 4.3. Comparative Experiment

In this section, several state-of-the-art methods based on deep learning are considered to conduct comparisons. These methods are first briefly introduced. Both $S_{32\times 32}$ and $S_{64\times 64}$ are considered in this experiment. Experimental results are presented in Table 2 and the best results are bold.

1. To identify CGs, Yao et al. [20] designed a CNN consisting of 5 convolutional modules (referred to as NoiseNet), where each convolutional module contains a convolutional layer, a batch normalization layer, ReLU and a pooling layer. NoiseNet applied three types of high-pass filters to extract residual signals (e.g., sensor noise) of the input samples before feeding them into the network.
2. In [21], Quan et al. proposed a CNN to detect CGs by stacking several convolutional modules (referred to as CGNet) where this CNN is initialized with a trainable convolutional layer, named as “convFilter”. Different from [20], which processes the input samples based on the prior knowledge of multimedia forensics, experimental results show that “convFilter” in CGNet can extract the band-pass frequency information for CG identification efficiently. It has the promising detection performance for image patches with various sizes.
3. He et al. [22] proposed a detection framework combining the convolutional and recurrent neural network to model the local and global statistics of CGs. In our experiments, the number of local patches is set as 4 = 2 × 2 and other settings are identical to those in [22]. We evaluate the performance of [22] using the samples with the resolution 64 × 64, since the method in [22] is invalid to conduct detection on very small image patch, such as 32 × 32.
4. Zhang et al. [23] proposed a convolutional neural network with channel and pixel correlation (referred to ScNet) to distinguish PGs and CGs. ScNet contains several hybrid correlation modules to enhance the discrimination capacity of CGs. In our experiments, we apply the ScNet in [23] to conduct detection directly for 64 × 64 image patches. On the other hand, for image patches with the smaller size (e.g., 32 × 32), we remove the last three convolutional modules in ScNet in [23] to construct the modified version of ScNet (more shallow) aiming to mitigate the overfitting.

As shown in Table 2, the proposed method can achieve the best detection performance compared with other state-of-the-art methods for image patches with the small size. It can be observed that with the decrement of image size, distinguishing CGs and PGs becomes more challenging due to the more limited information within one image patch. For the size of 64 × 64, the proposed method outperforms NoiseNet, CGNet, ScNet and HeNet by 8.63%, 5.81%, 5.19% and 5.25%, respectively. It implies that the proposed network which jointly optimizes raw RGB components and their blurred versions with the attention-based dual-branch CNN can extract more reliable deep representations for CG identification. On the other hand, existing deep learning-based methods that simply stack convolutional layers or apply manually designed high-pass filters to extract residual signals as input have a higher risk of overfitting. In practical applications, the more reliable detection capability of image patches with a small size is helpful to the detection of local tampering operations with CGs. Furthermore, the proposed network, ScNet and HeNet have a more balanced detection capability for positive samples (patches from CGs) and negative samples than CGNet and NoiseNet.

### 4.4. The Analysis of Different Network Architectures

In this work, we proposed an attention-based dual-branch network for CG identification. It is significant to evaluate how different parts of this network influence the detection performance, including different fusion strategies, different input components, different depths of each branch and network structures. In this section, only training and testing samples with 64 × 64 are considered ($S_{64\times 64}$).

#### 4.4.1. Different Fusion Strategies

In the proposed network, we consider both raw RGB components which have been used as the input for the CNN directly in [21,23] and blurred RGB components which are used to mitigate the overfitting problem and achieve better robustness against post-processing. It is significant to investigate which kind of fusion strategies is more suitable for these two types of components. In view of the network architecture, there are two candidates for fusion strategies, including early fusion strategy and late fusion strategy. The fusion strategy presented in Section 3.2.3 is early fusion strategy. On the other hand, in late fusion strategy, raw RGB components and their blurred versions are fed into two branches separately without stacking together and other network architectures keep unchanged. Experimental procedures are the same as the settings in Section 4.3. The results are reported in Table 3.

It can be observed that the early fusion strategy (Early Fusion (Attention) in Table 3) outperforms the late fusion strategy (Late Fusion (Attention) in Table 3) by more than 2%. It implies that the complementary relationship is exploited more efficiently by learning deep representations from raw and blurred RGB components which are stacked together in the early stage. On the other hand, learning from those two types of components individually in the late fusion structure is harder to jointly extract their low-level forensics traces. Furthermore, for early fusion structure, different fusion modules can be applied to fuse the output features from two branches, such as average fusion, concatenation fusion and attention-based fusion. The evaluation performance with different fusion modules is reported in Table 3.

As shown in Table 3, attention-based fusion module can achieve the best detection accuracy compared with other fusion modules, which can assign asymmetric weights to deep representations of different branches. It implies that deep representations learned by the branches with multi-scale convolutional kernels (including 3 × 3 and 5 × 5) perform different importance for various image contents. Please note the average fusion module can be regarded as a special case of attention-based fusion module where the weights assigned to output features of different branches are equal.

#### 4.4.2. Different Input Components

In several previous works, such as [21,23], the RGB components are fed into a neural network directly without any preprocessing operations to detect CGs. However, for image patches with the small size, the information in small patches is limited and diverse, which makes the network prefer to fit the details of local regions. Thus, we apply the early fusion strategy to stack raw and blurred RGB components in channel-wise as the input to mitigate the risk of overfitting and achieve better robustness against post-processing. In this experiment, the complementary relationship between raw and blurred RGB components is investigated. We consider three types of inputs, including (1) raw RGB components (Raw RGB), (2) blurred RGB components (Blurred RGB) and (3) stacking raw and blurred RGB components together (Raw + Blurred). Other network structures and experimental settings keep unchanged as Section 4.3. The results are presented in Table 4.

It can be observed that stacking raw RGB components and their blurred versions can improve the detection performance distinctly compared with only applying one type of components individually (more than 2%). Based on these results, blurred RGB components are efficient to help the network learn more generalized representations from spatial-wise and channel-wise correlation between different color components. It also implies that a suitable preprocessing operation is efficient to improve the performance of deep-learning-based CG identification algorithm, when the scale of training samples is relatively limited.

It is interesting to investigate how components in different frequency bands influence the detection performance of the proposed method. In this experiment, several widely-used post-processing operations are considered to process both training and testing samples, including bilateral filtering (with ($\sigma_{\mathrm{Color}},\sigma_{\mathrm{Space}}$) = (50, 50) and the default diameter), median filtering (with the operation window size equaling to 5×5), resizing (with the upscaling factor as 8% and then cropping the 64 × 64 central region). The above-mentioned post-processing operations are implemented based on OpenCV 3.4.1. Before being fed into the proposed network, the samples (raw RGB components), denoted as x, are processed by two kinds of preprocessing operations: (1) the Gaussian blurring operation with the same parameter setting in Section 3.2 to extract low frequency components, denoted as G(x), (2) extracting middle and high frequency components by the operation: x−G(x). Then, the preprocessed samples are fed into our proposed network. Other experimental procedures and settings were kept unchanged as in Section 4.3. The results are reported in Table 5.

As shown in Table 5, the proposed method considering low frequency components and middle/high frequency components can achieve the competitive performance (the difference of detection accuracies is less than 1%) when post-processing operations are not applied. It implies that the generation process of CG can leave distinguishable traces in both low-frequency components and middle/high frequency components (e.g., texture information). This result is in line with the conclusions in previous works [20,21]. On the other hand, the detect performance of the proposed method with middle/high frequency components suffers from a more severe performance drop compared with that of low-frequency components, especially for the median filtering operation (about 6%). It is because that the median filtering operation can disturb the local statistics dramatically. The experimental result demonstrates that the blurred version of raw RGB components is more reliable when the undesired distortions caused by post-processing operations exist. This advantage is important in realistic forensics analysis. Consequently, in our proposed method, we consider both raw RGB components and their blurred versions with an early fusion strategy to achieve both high detection capability and robustness against post-processing operations.

#### 4.4.3. Different Depths of the CNN Branch and Network Structures

CNN can benefit from increasing the depth of the network suitably. In this experiment, we investigate how the different depths of the CNN branch influence the detection performance of our proposed network. The proposed network is modified by adding a convolutional module after Conv3 which has the identical structure as Conv3 in Table 1 (referred to as Five Conv Modules) or removing Conv3 in the current network (referred to as Three Conv Modules). Other network structures and experimental procedures are kept unchanged as in Section 4.3. The detection results are reported in Table 6.

It can be observed that decreasing the depth of the CNN branch can cause performance drop about 1.2% due to the underfitting of the training samples. On the other hand, the detection performance of the proposed network with four and five convolutional modules are competitive. It implies that simply increasing the depth of the CNN branch is hard to improve the detection perform distinctly and may have a higher risk of overfitting. Considering both computational efficiency and detection performance, the proposed network contains four convolutional modules.

To verify the advantage of our proposed network structure, we conducted modifications on the proposed dual-branch CNN, including: (1) Setting the initial convolutional layers have the same kernel size (3 × 3 or 5 × 5) in both branches, (2) adding the shortcut connection [27] between convolutional layers. Other network architectures and experimental settings are kept unchanged as in Section 4.3. Experimental results are reported in Table 7.

As shown in Table 7, the proposed method can achieve the best detection accuracy compared with the modified versions. The poorer detection performance of modified versions may be due to the following reasons: (1) Two branches equipped with the identical structure are harder to extract deep representations in multi-scale which is useful to achieve better performance. (2) It cannot benefit from applying the shortcut connection for the task of CG identification which focuses on the low-level forensics traces. The shortcut connection [27] is originally designed to overcome the problem of vanishing/exploding gradients in networks with the deep architecture for computer vision tasks. In summary, our dual-branch network leverages the conventional convolutional layers and sets different kernel sizes in different branches to learn robust deep representations of low-level forensics traces in multi-scale. Experimental results in Table 7 demonstrate that the considerations of designing our network can lead to the better detection performance than other network structures.

### 4.5. The Robustness against Post-Processing Operations

In social media, digital images are always transmitted and stored in JPEG format due to the limitation of bandwidth and storage. Therefore, it is valuable to evaluate how the proposed method and other methods perform under JPEG compression especially for small image patches. In this experiment, samples in $S_{64\times 64}$ are compressed into JPEG format and the quality factors (QF) are selected from the set {95, 85, 75}. The quality factors below 75 are not considered since block artifacts are visually obvious in these cases due to lossy quantization in JPEG compression. Other experimental settings keep unchanged as Section 4.3. The results are presented in Table 8.

It can be observed that all of the methods suffer from the performance drop compared with the results without JPEG compression in Table 2. It is because the lossy quantization operation in JPEG compression can erase the details in samples which is useful to distinguish CGs and PGs. More specifically, ScNet suffers from the largest performance drop among all methods. It is because the design of ScNet considers the pixel correlation which is sensitive to lossy quantization. In addition, CGNet performs better than ScNet and NoiseNet. We conjecture that the band-pass information learned by “convFilter” in CGNet is more reliable after JPEG compression. The proposed method achieves the most robust performance against other methods. It demonstrates that the early fusion strategy of different color components combined with the proposed AD-CNN is efficient to learn more robust and generalized representations for CG identification with the small image patch. This advantage is significant in practical applications.

In practical applications, the forgers may adopt the post-processing operations to hide the forensics traces left by the generation process of CGs. It is significant to evaluate the robustness of the proposed method against different post-processing operations. In this experiment, several widely-used post-processing operations are considered to process both training and testing samples, including bilateral filtering (with ($\sigma_{\mathrm{Color}},\sigma_{\mathrm{Space}}$) ∈ {(25, 25), (50, 50)} and the default diameter), median filtering (with the operation window size equaling to {3×3,5×5}), resizing (with the upscaling factor as {4%, 8%} and then cropping the 64 × 64 central region), Gaussian noise addition (with zero mean and σ∈{0.8,1.2}), sharpening (the factor controlling the strength of sharpening operation is set as {0.5, 1}). The above-mentioned post-processing operations are implemented based on OpenCV 3.4.1. Other experimental setting and procedures keep unchanged as Section 4.3. Results are reported in Table 9.

As shown in Table 9, the proposed method still achieves the best detection performance compared with existing methods. It implies that the advanced techniques introduced in our method, including the early fusion of raw and blurred RGB components, dual-branch CNN with different kernel sizes in initial convolutional layers and the attention-based fusion module, can help to learn more robust deep representations against post-processing operations. It is interesting that NoiseNet [20] applying high-pass filters to obtain residual signals as input has a distinct performance drop with post-processing operations. It demonstrates that the high-frequency components are more sensitive and easier to be influenced by undesired distortions for distinguishing CGs and PGs.

## 5. Conclusions

In this work, we have proposed an attention-based dual-branch CNN (AD-CNN) to identify CGs with the small size from fused color components. For a given sample, the early fusion strategy is considered to stack raw RGB components and their blurred versions in channel-wise as the input of the network, which aims to help the network learn more robust representations within or cross different color components. The proposed AD-CNN contains two branches with an identical shallow CNN structure except for that multi-scale kernel sizes are adopted in the first convolutional layers of different branches. The output features from two branches are jointly optimized by the attention-based fusion model and followed by three fully-connected layers to obtain the final detection results. Extensive experiments demonstrate the better detection performance and robustness of the proposed method compared with state-of-the-art methods for CG identification with the small patch size and common post-processing operations. In addition, self-analysis experiments verify the significance of the advanced fusion strategies and network structures introduced in our proposed method, such as blurred RGB components and the attention-based fusion modules. In future work, we plan to extend our proposed method to conduct the localization of tampered regions created by CGs.

## Figures and Tables

**Table 1:** The detailed structures of each branch.

No.	Type	Settings
Conv 1	2D Convolution	[k,k,1,1,64] ^1^
	Batch Normalization	-
	ReLU	-
	Maxpooling	[3,3,2,2] ^2^
Conv 2	2D Convolution	[3,3,1,1,128]
	Batch Normalization	-
	ReLU	-
	Maxpooling	[3,3,2,2]
Conv 3	2D Convolution	[3,3,1,1,128]
	Batch Normalization	-
	ReLU	-
	Maxpooling	[3,3,2,2]
Conv 4	2D Convolution	[3,3,1,1,128]
	Batch Normalization	-
	ReLU	-
	Global Averagepooling	-

^1^ For the setting of 2D convolutional layer ([k,k,s,s,d]), k×k denotes the kernel size, s×s denotes the stride and *d* denotes the number of output feature maps. *k* equals 3 and 5 in the initial convolutional layer of the first and the second branch, respectively; ^2^ For the setting of maxpooling layer ([k,k,s,s]), k×k denotes the window size of the pooling operation and s×s denotes the stride.

**Table 2:** Performance comparisons with state-of-the-art methods (%).

	Performance	TNR	TPR	Acc
Patch Size/Methods				
32 × 32	NoiseNet [20]	78.90	72.16	75.53
	CGNet [21]	83.29	77.35	80.32
	ScNet [23]	79.86	78.56	79.21
	Proposed method	84.29	83.21	83.75
64 × 64	NoiseNet [20]	82.63	75.75	79.19
	CGNet [21]	84.61	79.41	82.01
	ScNet [23]	84.20	81.06	82.63
	HeNet [22]	83.65	81.49	82.57
	Proposed method	87.43	88.21	87.82

**Table 3:** Detection performance with different fusion strategies (%).

Fusion Strategies	TNR	TPR	Accuracy
Late Fusion (Attention)	84.60	87.01	85.81
Early Fusion (Concatenate)	85.71	86.43	86.07
Early Fusion (Average)	86.56	85.32	85.94
Early Fusion (Attention)	87.43	88.21	87.82

**Table 4:** Detection performance with different inputs (%).

Input Components	TNR	TPR	Accuracy
Raw RGB	85.22	85.80	85.51
Blurred RGB	83.79	85.95	84.87
Raw + Blurred	87.43	88.21	87.82

**Table 5:** The detection performance with different frequency components (%).

	Low Frequency Components	Middle and High Frequency Components
Without post-processing	84.87	83.91
Bilateral filtering	83.38	80.06
Median filtering	82.47	76.49
Resizing	83.96	81.14

**Table 6:** Detection performance with different depths of the convolutional neural network (CNN) branch (%).

Depths of the CNN Branch	TNR	TPR	Accuracy
Three Conv Modules	86.73	86.45	86.59
Five Conv Modules	87.59	88.09	87.84
Four Conv Modules	87.43	88.21	87.82

**Table 7:** The detection performance with different network architectures (%).

	Proposed	Modification 1 ^a^	Modification 2 ^b^	Modification 3 ^c^
Detection accuracy	87.82	86.41	86.15	86.33

^a^ Applying the same kernel size (3 × 3) in the first convolutional layers of both branches; ^b^ applying the same kernel size (5 × 5) in the first convolutional layers of both branches; ^c^ applying the shortcut connection.

**Table 8:** Detection accuracies of different methods after JPEG compression (%).

	Methods	Proposed Method	ScNet	CGNet	NoiseNet
Quality Factor					
95		86.90	81.25	80.13	78.29
85		83.51	73.38	77.15	71.21
75		81.88	71.51	74.96	70.35

**Table 9:** The detection performance against different post-processing operations (%).

	Bilateral Filter		Resizing		Median Filter		Sharpening		Noise Addition	
Parameter	(25,25)	(50,50)	4%	8%	3 × 3	5 × 5	0.5	1	0.8	1.2
NoiseNet	73.79	72.35	75.96	75.22	73.31	70.32	76.94	76.12	77.24	76.03
CGNet	80.48	80.20	81.62	80.00	79.04	76.49	81.03	80.18	81.47	80.86
ScNet	78.27	77.95	80.76	80.21	78.12	72.57	80.64	79.38	81.53	81.05
HeNet	80.40	79.74	81.21	80.58	80.43	77.86	81.52	80.90	81.13	80.54
Proposed	86.15	85.78	86.69	86.21	85.79	83.24	86.81	86.28	87.23	86.85
